# Exploring the correlation between the sequence composition of the nucleotide binding G5 loop of the FeoB GTPase domain (NFeoB) and intrinsic rate of GDP release

**DOI:** 10.1042/BSR20140152

**Published:** 2014-12-12

**Authors:** Amy P. Guilfoyle, Chandrika N. Deshpande, Gerhard Schenk, Megan J. Maher, Mika Jormakka

**Affiliations:** *Structural Biology Program, Centenary Institute, Locked Bag 6, Sydney, New South Wales 2042, Australia; †Faculty of Medicine, Central Clinical School, University of Sydney, Sydney, New South Wales 2006, Australia; ‡School of Chemistry and Molecular Biosciences, The University of Queensland, St. Lucia, Queensland 4072, Australia; §La Trobe Institute for Molecular Science, La Trobe University, Melbourne, Victoria 3086, Australia

**Keywords:** crystal structure, GDP release, GTPase, sequence motif, stopped flow, GPCR, G protein-coupled receptor, *Ec*NFeoB, *Escherichia coli* FeoB, *St*NFeoB, *Streptococcus thermophiles* FeoB, TEV, tobacco etch virus

## Abstract

GDP release from GTPases is usually extremely slow and is in general assisted by external factors, such as association with guanine exchange factors or membrane-embedded GPCRs (G protein-coupled receptors), which accelerate the release of GDP by several orders of magnitude. Intrinsic factors can also play a significant role; a single amino acid substitution in one of the guanine nucleotide recognition motifs, G5, results in a drastically altered GDP release rate, indicating that the sequence composition of this motif plays an important role in spontaneous GDP release. In the present study, we used the GTPase domain from *Ec*NFeoB (*Escherichia coli* FeoB) as a model and applied biochemical and structural approaches to evaluate the role of all the individual residues in the G5 loop. Our study confirms that several of the residues in the G5 motif have an important role in the intrinsic affinity and release of GDP. In particular, a T151A mutant (third residue of the G5 loop) leads to a reduced nucleotide affinity and provokes a drastically accelerated dissociation of GDP.

## INTRODUCTION

GTPases are involved in essential cellular processes in both prokaryotes and eukaryotes. When bound to GTP, GTPases are in their ‘active’ or signal transducing state, where reversible conformational changes allow for interaction with effector molecules and regulation of cellular functions [1]. Upon either intrinsic or catalysed hydrolysis, inorganic phosphate is released and the resulting GDP–GTPase complex reverts into an ‘inactive’ state [2,3]. Central to this cyclic process is the release of GDP, which allows for GTP and effector molecules to bind. However, GTPases have in general a high affinity for GDP (in the nano- to low micromolar range), resulting in an extremely slow spontaneous dissociation rate [4,5]. To accelerate the dissociation rate, many systems require guanine exchange factors, which catalyse the release of GDP by several orders of magnitude [2,4,6].

**Figure 1:**
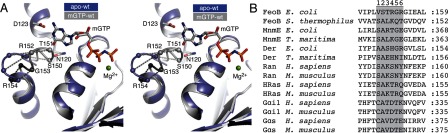
Structural changes and sequence alignment of the G5 loop (**A**) Stereo view of the G5 loop in *Ec*NFeoB. Nucleotide free (PDB code 3HYR) and nucleotide bound (3HYT) structures are shown in purple and grey, respectively. The residues in the G5 loop and selected residues involved in nucleotide base coordination are labelled and shown as spheres and ball-and-stick, respectively. The conformational shift of individual Cα atoms in the G5 loop is illustrated with dotted lines. GMPPNP is shown as ball-and-stick, with the mant group removed for clarity. (**B**) Sequence alignment of the residues in and around the G5 sequence motif. Residues numbered and shaded in grey are, in this study, designated as G5 loop residues.

In eukaryotic heterotrimeric Gα(βγ) proteins, the interaction with their cognate membrane-bound GPCR (G protein-coupled receptor) catalyses the release of GDP [7]. Although some details remain unknown, studies using double electron–electron resonance, deuterium-exchange, Rosetta energy analysis and electron paramagnetic resonance, have shown that the association between a Gα and its cognate GPCR catalyses the release of the nucleotide by, at least in part, causing a structural change in the G5 sequence motif, one of five guanine nucleotide binding motifs (G1–G5) [7–14]. Structural studies have also illustrated a large conformational change in the G5 loop of the intracellular Ras-type GTPase domain (NFeoB) of the prokaryotic iron transporter FeoB, when comparing crystal structures of apo and nucleotide bound states (Figure 1A) [15,16].

The G5 motif is generally comprised of six amino acids with a relatively poor sequence conservation, although the motif invariably forms a loop with electrostatic or hydrophobic interactions with the guanine nucleotide base (Figure 1) [1,3,17]. Despite the lack of overall sequence conservation, several studies have shown that the sequence composition of the G5 loop plays an important role in influencing the intrinsic or spontaneous GDP release rate; the second residue of the motif is generally conserved as an alanine (Figure 1B), and mutation to this residue in the human Gα_s_- protein (A366S) results in a constitutively active state through a drastically accelerated GDP release, causing testotoxicosis and pseudohypoparathyroidism in young men [18]. In addition, mutation to the equivalent residue in human HRas leads to rapid GDP release rates, is clinically manifested as the Costello syndrome [19,20] and is also present at high frequency in colorectal cancer [21,22]. A recent mutational analysis of the equivalent residue in the prokaryotic NFeoB from *St*NFeoB, *Streptococcus thermophilus* FeoB also illustrated similar acceleration of the GDP release rate [23]. To understand the mechanism of the accelerated release, crystal structures of a Gα*_i_*_1_(β_1_γ_2_) A326S mutant and a *St*NFeoB A143S mutant (both equivalent to the Gα_s_ A366S mutation) in complex with GDP were determined. These structures revealed that the mutation at this position of the G5 loop causes a displacement of the nucleotide base, an altered hydrogen-bonding network, and reduced GDP affinity [23,24].

Although substantiating the relevance of the G5 sequence composition in GDP release, these studies have exclusively been focused on the second residue of the motif. Here, we seek to expand on previous studies by analysing the significance of each residue of the G5 loop and the correlation between sequence and the intrinsic rate of GDP release. We have used the *Ec*NFeoB (*Escherichia coli* FeoB) protein as a model system because of its clearly resolved conformational change in the G5 loop as revealed by crystal structure analyses (Figure 1A) [16]. We systematically mutated the residues in the loop to alanine and characterized their biochemical properties in order to determine their individual roles in nucleotide affinity, GTP hydrolysis and GDP release. Our studies reveal that the intrinsic GDP release rate is highly influenced by the sequence composition of the loop, with the third residue (T151) in particular having an important role in nucleotide affinity.

## MATERIALS AND METHODS

### Protein preparation and mutagenesis

The DNA encoding residues 1–270 of FeoB was amplified from *E. coli* K12 genomic DNA and cloned into the pGEX-4T-1 expression vector (GE Healthcare). The protein was expressed in BL21 (DE3) cells as a GST fusion construct and purified as previously described in [16]. In brief, the expressed protein was purified by GST affinity chromatography (GE Healthcare) and the GST moiety was subsequently removed by thrombin cleavage at 30 °C over 48 h. The protein was further purified by size exclusion chromatography (Superdex 75, GE Healthcare), after which the protein was buffer exchanged into Tris pH 8.0 (20 mM), concentrated to approximately 10 mg ml^−1^ and stored at −80 °C until further use. Single amino acid substitutions in the G5 loop motif (V149A, S150A, T151A, R152A, G153A and R154A) were generated using the wild-type NFeoB as template and site-directed mutagenesis (QuikChange II XL, Stratagene). In addition, an alternate construct, termed S150A-TEV (tobacco etch virus), for the S150A *Ec*NFeoB mutant was cloned into the expression vector pGEX-4T-1 using the restriction sites BamHI and XhoI. The forward primer (5′-GGGGGATCCGAAAACCTGTACTTCCAGGGTCAATTC-ATGAAAAAATTAACCATTGGC) included the sequence for a TEV (shown underlined) protease recognition site for downstream GST affinity tag removal. Expression and purification was carried out using the protocol described for the wild-type protein, except with the removal of the GST moiety carried out using TEV protease (0.5 mg ml^−1^) at 4 °C overnight.

### GTPase activity measurements

As *St*NFeoB was previously shown to have an accelerated GTP hydrolysis rate in the presence of K^+^ [15], we initially measured the GTP hydrolysis rate for the wild-type NFeoB under varying salt conditions using a malachite green phosphate assay (BioAssay Systems). Protein (0.3 μM) was incubated with GTP (400 μM) and MgCl_2_ (5 mM) in Tris pH 8.0 (20 mM) with salt (200 mM KCl, LiCl, NaCl or NH_4_Cl) at 30 °C. Hydrolysis proceeded for an average of 3.5 h with aliquots removed at frequent intervals and mixed with the malachite green reagent in a 4:1 ratio as per manufacturer specifications. Colour was developed for 30 min at room temperature prior to absorbance measurements at 620 nm on a POLARstar Omega microplate reader (BMG Labtech) in a 96-well plate (Greiner Bio-One). The hydrolysis rates of the G5 loop mutants were determined in the presence of K^+^ (200 mM). The enzyme turnover number (*k*_cat_) was additionally determined by means of linear regression for wild-type and mutant NFeoB at 37 °C in conditions outlined above using KCl salt only. All hydrolysis assays were performed in triplicate.

### Stopped-flow fluorescence assays

The binding and release rates of fluorescent nucleotides by wild-type *Ec*NFeoB and G5 loop mutant proteins were analysed using stopped-flow fluorescence assays. To determine release rates (*k*_off_), the protein (10 μM) was incubated with the fluorescent nucleotide mant-GDP (0.5 μM), in stopped-flow buffer (20 mM Tris pH 8.0, 100 mM NaCl and 100 mM MgCl_2_) for 30 min at room temperature. Equal volumes of the protein–mant-GDP mix and GTP (1 mM) in stopped-flow buffer were rapidly mixed into a 100 μl optical cell of a pneumatically driven stopped-flow apparatus (SMV-17MV, Applied PhotoPhysics). The mant group was excited at 360 nm and fluorescence was monitored through a 405 nm cut-off filter. Similarly, nucleotide-binding rates (*k*_obs_) were determined by rapidly mixing protein (2.5–80 μM) with the fluorescent, non-hydrolysable GTP analogue mant-GMPPNP (1 μM) in the stopped-flow apparatus. All data reported are averaged from 7 to 10 independent experimental traces performed under identical conditions. Reactions were performed at 20 °C.

### Isothermal titration calorimetry (ITC)

ITC was used to measure the GDP-binding affinities of wild-type *Ec*NFeoB and mutant proteins. Protein (approximately 0.1 mM) in buffer (20 mM Tris pH 8.0 and 100 mM NaCl) was equilibrated for 1 min at 25 °C with stirring (1000 rpm) in the sample cell of a MicroCal iTC_200_ Isothermal Titration Calorimeter. GDP (2.5–5 mM) was titrated into the sample cell in 0.5–2 μl injections over 0.8 s with 150 s spacing between injections. Power input (μcal s^−1^) required to maintain equal temperatures between the sample and reference cells in response to each addition of ligand was plotted versus time (min). The data were integrated and plotted versus the molar ratio of ligand to protein. Non-linear regression was used to obtain the thermodynamic parameters (including GDP-binding affinity, *K*_a_). Data were fitted to a one-site binding model using the Origin 7 Software (MicroCal) to obtain stoichiometry (N), enthalpy (ΔH), entropy (ΔS) and association rate constant (*K*_a_). The dissociation constant (*K*_d_) was calculated from Equation 1 (*K*_d_=1/*K*_a_). All reported values are the average of three or more independent titrations. Due to interdiffusion of the solutions during the insertion of the syringe into the sample chamber, the first injection is not useful for analysis and was omitted from all calculations.

### Protein crystallization

To validate the structural integrity of the mutants and to obtain further insight into their role in GDP release, we pursued structural studies of the mutant proteins with altered biochemical characteristics. Crystals of apo-S150A protein were grown in drops containing a one-to-one ratio of protein with reservoir solution containing 2% PEG 400, 0.2 M (NH_4_)_2_SO_4_, 0.1 M Bis-Tris pH 5.5 and 25% PEG 3350, using the hanging drop technique. Microcrystals appeared overnight at 20 °C, which grew to maximum size (0.2 mm×0.3 mm) in 2 months. The apo-T151A protein (10 mg ml^−1^, 20 mM Tris pH 8.0) was crystallized by vapour diffusion using the hanging drop technique, by mixing the protein in a two-to-one ratio with reservoir solution at 20 °C. Optimized crystals grew to maximum dimensions of 0.4 mm×0.4 mm×0.2 mm at 20 °C using 29% PEG 400, 0.1 M Hepes pH 7.8 as a reservoir solution. Data processing, model building and refinement are described in Supplementary Material, and are summarized in the Supplementary Table S1. Coordinates and structure factors were deposited to the Protein Data Bank under codes 4Q00 (apo-S150A) and 4Q5I (apo-T151A).

## RESULTS AND DISCUSSION

### GTPase activity in monovalent salts

In this study, we defined the G5 loop as the residues included in the conformational change when comparing the structures of apo and nucleotide bound *Ec*NFeoB (Figure 1A), which corresponds to residues V149–G154 (Figure 1B). A suite of single amino acid substitutions of these residues was generated through site-directed mutagenesis, and with the exception of the V149A mutant (insoluble) we could purify all mutants to homogeneity. GTPase activities of purified proteins were subsequently estimated in steady state assays using the malachite green assay (BioAssay Systems). Our previous studies of *St*NFeoB [15] revealed a K^+^ dependent activation of the GTP hydrolysis, which has now been recognized as a signifying characteristic in the TEES superfamily of GTPases [25]. To confirm that this is also the case for *Ec*NFeoB, we performed GTP hydrolysis measurements of wild-type protein in the presence of a range of monovalent salts (Supplementary Figure S1). The order of activity in the different salts was KCl>NH_4_Cl>LiCl>NaCl, consistent with the previous studies of orthologous proteins in the TEES superfamily of GTPases [15,26]. The hydrolysis rate for the wild-type *Ec*NFeoB protein in the presence of K^+^ ions was approximately seven times faster than the measured rates in the presence of Na^+^ ions (*k*_cat_=0.40 min^−1^ versus 0.06 at 30 °C). Earlier studies of the orthologous protein from *S. thermophilus* illustrated 20-fold acceleration in the presence of K^+^ ions versus Na^+^ ions [15]. Although acceleration of the hydrolysis rate in *Ec*NFeoB is not as dramatic, it still clearly illustrates a K^+^-dependent activation of hydrolysis. The enzyme turnover rates (*k*_cat_) for wild-type and mutant proteins were subsequently determined at 37 °C in the presence of 200 mM KCl (Table 1 and Supplementary Figure S1). Under experimental conditions, the S150A mutant demonstrated the fastest GTP hydrolysis rate (*k*_cat_=0.67 min^−1^), a rate approximately 1.5 times faster than that of wild-type *Ec*NFeoB. All other mutants were determined to have slower hydrolysis rates than the wild-type protein, with the T151A mutant being approximately four times slower than the wild-type protein (*k*_cat_=0.12 min^−1^; Table 1 and Supplementary Figure S1).

**Table 1:** Enzyme turnover rates, stopped-flow data

Sample	*k*_cat_ (min^−1^)*	*k*_on_^†^ (μM^−1^ min^−1^) GMPPNP	*k*_off_^‡^ (min^−1^) GMPPNP	*K*_d_^§^ (μM) GMPPNP	*k*_off_^∥^, ^¶^ (s^−1^) GDP	*K*_d_^**^ (μM) GDP
**NFeoB**	0.46±0.06	8.1±0.1	79±2	9.7	145±2	9±1
**S150A**	0.67±0.06	26±1	0±0^††^	0	22±1	2±0.3
**T151A**	0.12±0.04	5.9±0.3	159±5	27	n.d^‡‡^	33±4
**R152A**	0.35±0.03	14±1	314±5	23	100±3	8±1
**G153A**	0.26±0.03	6.7±0.3	115±1	17	193±8	11±2
**R154A**	0.17±0.03	4.3±0.1	184±1	43	133±4	9±2

*k_cat_ was determined for target proteins in 200 mM KCl at 37 °C. Results were averaged from three or more experiments using a malachite green phosphate assay.

^†^*k*_on_ was determined from the slope of the linear plot formed by *k*_obs_ at protein concentrations between 1.25 and 40 μM;

**^‡^***k*_off_ was determined from the *y*-intercept of the linear plot.

^§^*K*_d_ was determined from the ratio of *k*_off_ to *k*_on_.

^∥^mGDP dissociation rates (*k*_off_) for *Ec*NFeoB and mutants were determined by fitting a single exponential function to stopped flow data.

^¶^*k*_off_ values are the average of three or more stopped flow experiments with each experiment consisting of five or more replicates.

^**^K_d_ was determined for GDP using ITC.

^††^Under experimental conditions, the off rate was too low to be reliably determined.

^‡‡^Under the experimental conditions, the GDP release was too rapid to be reliably determined using stopped-flow.

### Nucleotide binding and release characteristics measured using stopped-flow fluorescence and ITC

**Figure 2:**
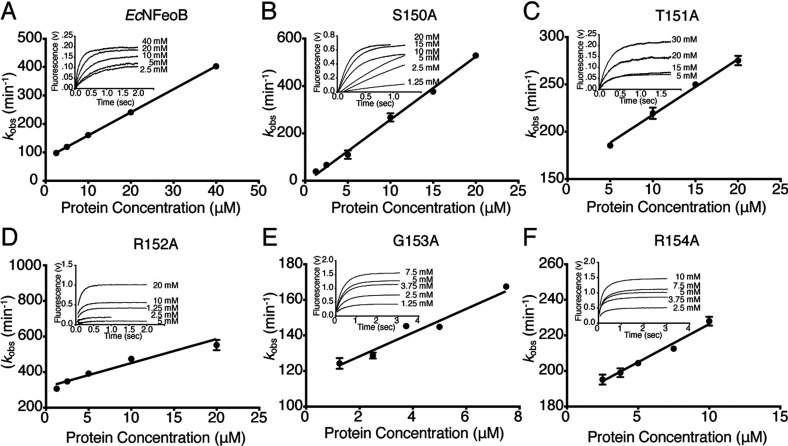
Stopped-flow analyses of GMPPNP binding Determination of the on-rate constants (*k*_on_) for mant-GMPPNP with (**A**) wild-type and (**B**–**G**) mutant *Ec*NFeoB proteins. Figure shows observed association rate constants (*k*_obs_) for mant-GMPPNP with *Ec*NFeoB proteins versus concentration. (Insert) Traces of mant-GMPPNP binding at the indicated final protein concentrations. Observed rate constants (*k*_obs_) were determined from the single exponential taken from each trace and plotted as a function of protein concentration. The observed rate constants have a linear relationship from which *k*_on_ can be determined.

We used stopped flow fluorescence assays to determine mant-GMPPNP association (*k*_on_) and mant-GDP dissociation (*k*_off_) rates for wild-type and mutant *Ec*NFeoB proteins. For each protein, the observed rate constants (*k*_obs_) were plotted versus protein concentration and the association rate constants (*k*_on_) were determined from the slope of the linear plots (Figure 2). The wild-type *Ec*NFeoB *k*_on_ value of 8.1±0.1 μM^−1^ min^−1^ was in agreement with the results of previous measurements [16]. The *k*_on_ values for the mutant proteins were variable but within the same order of magnitude, with the S150A mutant having the fastest association rate (26±1 μM^−1^ min^−1^) and the R154A mutant the slowest (4.3±0.1 μM^−1^ min^−1^; see Table 1).

**Figure 3:**
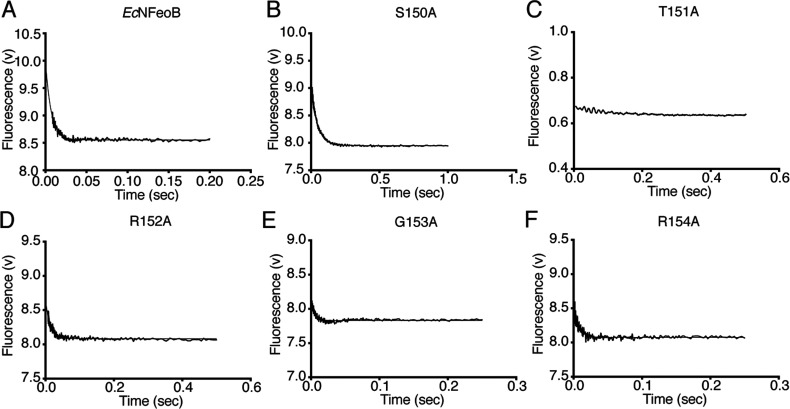
GDP release using stopped-flow and mant-GDP Dissociation of mant-GDP from (**A**) wild type and (**B**–**G**) mutant *Ec*NFeoB proteins. The off-rate constant (*k*_off_) was determined directly from the single exponential function of the data (see Table 1). Final *k*_off_ values are the average of three or more stopped-flow experiments with each experiment consisting of five or more replicates.

**Figure 4:**
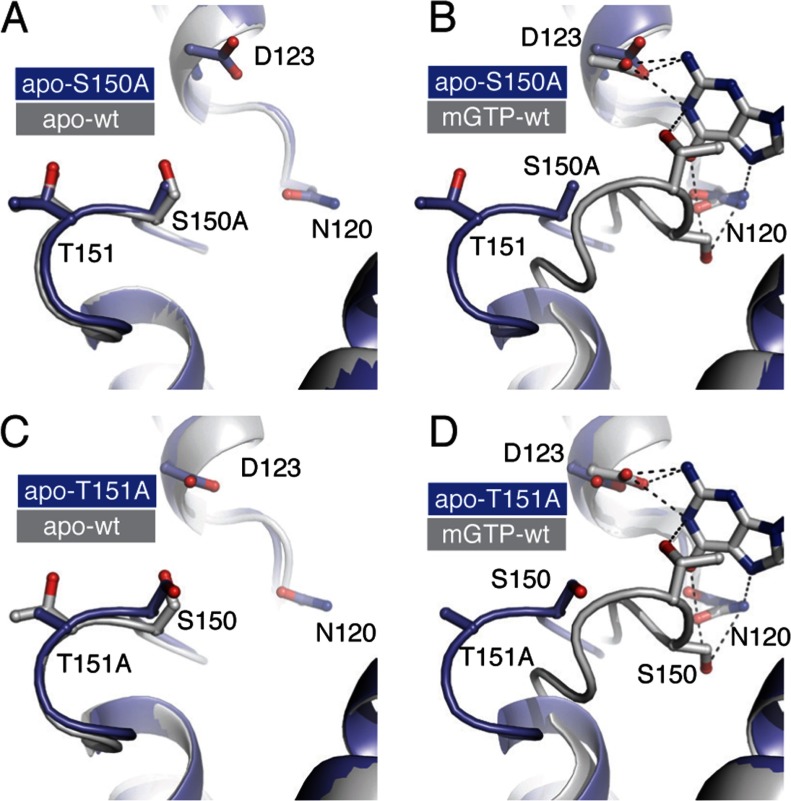
Structural validation and analysis of the S150A and T151A mutant proteins Structural overlay of the G5 loop of the S150A mutant with that of the (**A**) apo-wild type *Ec*NFeoB (PDB ID 3HYR) and (**B**) mGTP bound wild type *Ec*NFeoB (PDB ID 3HYT). The G5 loop of the S150A mutant is identical to the wild-type apo structure conformation and distinct from the nucleotide bound conformation. Bonds are shown as dotted line. (**C**,**D**) Structural overlay of the G5 loop of the T151A mutant with that of (C) apo-wild-type *Ec*NFeoB and (**D**) mGTP-bound wild-type *Ec*NFeoB. As with the S150A mutant structure, the T151A mutant structure is virtually identical in conformation to the wild-type apo *Ec*NFeoB structure. The electrostatic or Van Der Waal interactions in the wild-type structure highlight the interactions between the T151 hydroxyl group with the N1 nitrogen of the nucleotide, and the hydrophobic interaction between the T151 methyl group and the nucleotide base, which are lost with the T151A mutation.

FeoB from *E. coli* has been recognized as having an extremely fast intrinsic GDP dissociation rate [16,27], a characteristic shared with many other prokaryotic GTPases [28–30]. Here we determined the mant-GDP dissociation rate, *k*_off_, by fitting a single exponential function to the stopped-flow data (Figure 3). For wild-type *Ec*NFeoB the *k*_off_ was found to be 145±2 s^−1^, also consistent with the previously published rate [16,27]. In contrast, the *Ec*NFeoB G5 mutant proteins illustrated drastic variations in their GDP release rates. The R152A, G153A and R154A mutations did not alter the release rate significantly, while the S150A mutant has a release rate that is approximately 7-fold slower (*k*_off_=22.4±0.1 s^−1^) than that of the wild-type protein (Table 1). Interestingly, the release rate of the T151A mutant was too rapid (>1,000 s^−1^) to be reliably determined using stopped-flow fluorescence techniques. Given their drastic biochemical deviations, we also validated the integrity of the S150A and T151A mutants by determining their crystal structures, which illustrated that the mutations did not have any adverse effects on the overall fold of the protein or on the G5 loop (Figure 4 and Supplementary Material).

**Figure 5:**
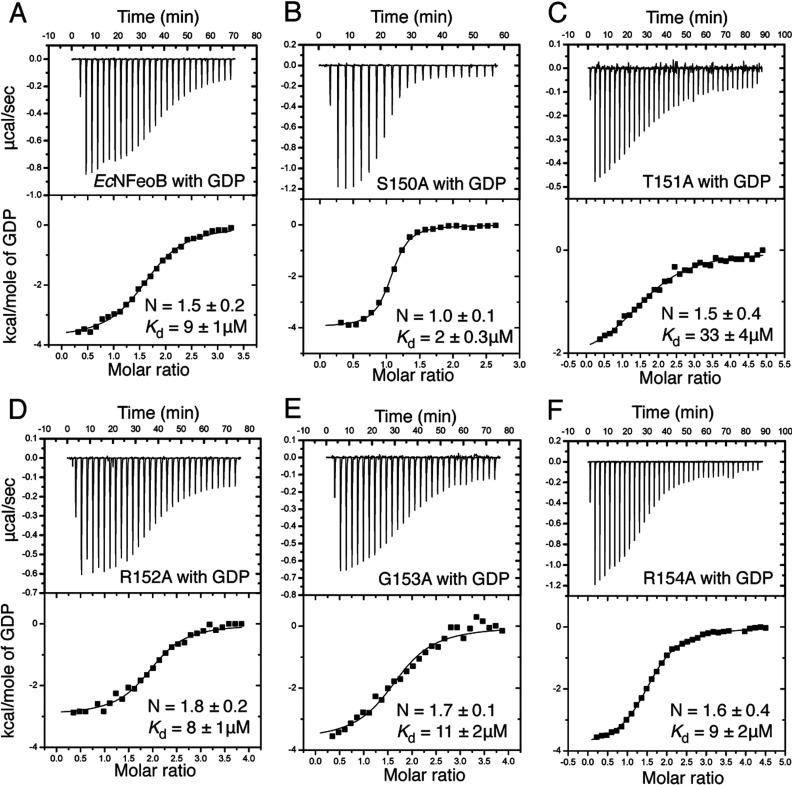
Thermodynamic binding assay ITC titration curves (upper) and binding isotherms (lower) for (**A**) wild-type and (**B**–**G**) mutant *Ec*NFeoB proteins interacting with GDP.

To substantiate the stopped flow results, we subsequently used ITC to measure the *K*_d_ values of GDP for wild-type and mutant *Ec*NFeoB proteins (Figure 5). The *K*_d_ values were calculated using the formula *K*_d_=1/*K*_a_, with the *K*_a_ values obtained from non-linear regression of integrated data. The ITC measurements revealed wild-type *Ec*NFeoB to have a GDP affinity similar to that of other prokaryotic GTPases (*K*_d_=9.1 μM) [28–30], although this affinity is approximately 10-fold weaker than that of *St*NFeoB [15]. In agreement with the stopped-flow studies, the R152A, G153A and R154A mutant proteins illustrated affinities similar to that of wild-type protein (Figure 5). Furthermore, the ITC experimental results showed that the S150A mutant protein had the greatest GDP affinity (*K*_d_=2.0 μM), approximately five times that of wild-type *Ec*NFeoB, which is also in agreement with a decelerated GDP release rate (Figure 3 and Table 1). These molecular phenotypes conform to previous studies of *Ec*NFeoB [16], as well as with studies of the equivalent residue in *St*NFeoB [23], Gα_s_ [18] and Gα*_i_*_1_ [24]. The rationale for the altered nucleotide-binding and release properties can be inferred from our recent studies of the inverse mutant of *St*NFeoB [23]. The *St*NFeoB protein has, as most other GTPases, an alanine residue at this position (Figure 1B). Comparing the structures of GDP bound wild-type *St*NFeoB and an A143S mutant protein (equivalent to *Ec*NFeoB S150), illustrated that the mutation destabilizes the nucleotide base by altering the hydrogen-bonding network. It is thus likely that this is also the case for the wild-type *Ec*NFeoB protein, while the S150A mutation generates a more stable nucleotide-binding environment.

### The third residue (T151) of the G5 loop significantly affects the intrinsic GDP release rate

The most drastic biochemical changes were observed for the T151A mutant protein. In the native nucleotide-bound *Ec*NFeoB structure (PDB code 3HYT), the T151 hydroxyl group forms an electrostatic interaction with the N1 nitrogen atom of the nucleotide base, while the methyl group is involved in hydrophobic interactions with the base (Figure 4C). A threonine residue is also found at this position in many Gα proteins where it coordinates the nucleotide base in an identical way. These stabilizing interactions are abolished in the T151A mutant, resulting in a ~4-fold decreased GDP affinity (*K*_d_=33 μM) and a GDP release rate too rapid to be reliably measured using stopped flow methods (>1000 s^−1^). Indeed, the stopped-flow fluorescence data indicate that mGDP is spontaneously released even before the competing nucleotide is added to the reaction (Figure 3C). The data also indicate a weaker affinity for GMPPNP, which is further supported by the reduced hydrolysis rate (Table 1 and Supplementary Figure S1). These drastic effects were unexpected given the lack of sequence conservation at this position, although it is notable that the residue at this position is, as far as we know, never found as an alanine or glycine residue. This is likely due to an absolute requirement for stabilizing the nucleotide base by providing either electrostatic or hydrophobic interactions (or both). Corroborating the wider implication for other GTPases, when the equivalent residue in HRas is mutated to an alanine (K147A), the fraction of GTP-bound HRas is drastically increased, indicating an acceleration of the GDP release rate [31].

### Clues to the GDP release mechanism

The G5 loop has been speculated to play a critical role in GTPase activation (i.e. structural changes enabling GDP release), particularly in Gα proteins [8,9,12,16,18,24,32]. Structural studies of *E. coli*, *S. thermophilus*, and *Klebsiella pneumoniae* NFeoB proteins have also illustrated a conformational change in the G5 loop when comparing nucleotide bound and nucleotide-free proteins (Figure 1A) [15,16,33,34]. However, one critical question remains: is it the loop movement that drives GDP release, or is the movement a consequence of release? Our results show that the two most important residues for intrinsic affinity and release of GDP are the second (S150) and third (T151) residues of the G5 loop, which are also the two residues exhibiting the largest conformational shift in *Ec*NFeoB (Figure 1A; 9 and 7 Å, respectively). A recent study using Rosetta energetic analysis also illustrated that the largest destabilization in the G5 loop of Gα*_i_*βγ upon receptor binding (i.e. GDP release) occurred at residues A326 and T327 (equivalent to S150 and T151 in *Ec*NFeoB) [8]. The shift in the nucleotide-binding threonine residue is particularly relevant, given that our results illustrate a strong correlation between the presence (T151) or absence (T151A) of stabilizing interactions and GDP release rate. This suggests that a GDP release mechanism involving a conformational change of the G5 loop (and thus the removal of the stabilizing interactions) would significantly reduce the affinity for GDP, and conceivably be sufficient for catalysing nucleotide release. Our results thus provide a biochemical rationale to the conceptual mechanism of G5 loop movement being the driving factor for GDP release.

## Online data

Supplementary data
