# Supplementary material for: Exploring the correlation between the sequence composition of the nucleotide binding G5 loop of the FeoB GTPase domain (NFeoB) and intrinsic rate of GDP release
Source: Biosci Rep. 2014 Dec 12;34(6):e00158. doi: 10.1042/BSR20140152 (PMC4266920; doi:10.1042/BSR20140152)
Supplement: Supplementary data [file bsr034e158ntsadd.pdf]

Supplementary Material

Exploring the correlation between the sequence composition of the nucleotide binding G5 loop of the FeoB GTPase domain (NFeoB) and intrinsic GDP release rate

Guilfoyle *et al.*

I. **Supplementary Figure**

II. **Supplementary Table**

III. **Supplementary Methods & Discussion**

II. Supplementary Figure

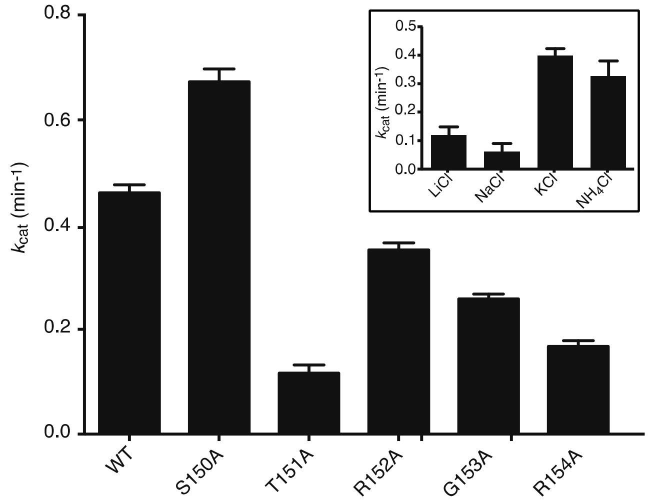

Supplementary Figure S1 - Hydrolysis rates of wild type and mutant proteins.
Determination of the enzyme turnover rate **(**k_{cat}**)** for GTP hydrolysis by wild type and mutant proteins at 37 °C. Protein was added to a solution containing GTP and $MgCl_2$. The release of inorganic phosphate was measured via a Malachite green colourimetric assay and plotted versus time. The enzyme turnover rate was measured by means of linear regression from the plot. (Inset) The hydrolysis rate for wild type *Ec*NFeoB protein in the presence of various monovalent salts was determined at 30 °C. All measurements were repeated in triplicates.

II. Supplementary Table

Supplementary Table SI. Data processing and refinement statistics for mutant *Ec*NFeoB proteins.

	S150A	T151A
Data collection		
Space group	$P2_1$	$P1$
Cell dimensions		
a, b, c (Å)	74.58, 56.16, 91.92	60.98, 82.78, 95.51
α, β, γ (deg)	90, 92, 90	116, 109, 90
Wavelength (Å)	0.953	1.03
Resolution (Å)[a]	47.97-2.10 (2.15-2.10)	45.06-2.80 (2.87-2.80)
R_{merge}[a]	0.105 (0.597)	0.066 (0.367)
$I/\sigma I$	9.2	11.2
Completeness (%)[a]	99.3 (98.6)	97.2(96.9)
Redundancy[a]	3.6 (3.5)	1.9 (1.5)
Wilson B factor ($Å^2$)	29.2	68.6
Refinement		
PDB Code	4Q00	4Q5I
Resolution (Å)	2.1	2.8
No. reflections	44446	35703
R_{work}/R_{free}	0.204/0.248	0.235/0.295
Residues included in the final model		
No. atoms		
Protein	5802	1584
Ligand/ion	15	5
Water	358	57
B-factors		
Protein	29.17	70.00
Ligand/ion	45.70	100.2
Water	34.40	52.3
r.m.s.d.		
Bond lengths (Å)	0.005	0.004
Bond angles (deg)	0.954	0.701
Ramachandran analysis[b]		
Favoured regions (%)	98.28	96.3
Allowed regions (%)	1.72	3.10

[a]Values in parentheses are for the highest-resolution shell.
[b]As assessed by MOLPROBITY .

III. Supplementary Methods & Discussion

Data Collection, Structure Determination, and Refinement

All crystals were flash frozen directly into a cold N_2 gas stream (100 K). S150A crystals were cryoprotected in a solution comprising 1:1 ratio of protein storage solution and crystal condition with the addition of 25 % glycerol and 2 % ethanol. T151A crystals were frozen without additional cryoprotection. X-ray diffraction data for S150A crystals were collected using an ADSC QUANTUM 315r detector at beamline MX2 of the Australian Synchrotron. The data was processed and scaled using Mosflm [1] and Scala from the CCP4 program suite [2], respectively. Diffraction data for T151A crystals was collected using a MARmosaic 300 detector at beamline ID23-D at the Advanced Photon Source (APS, Chicago). Data was processed and scaled using HKL2000 [3]. Both structures were solved by molecular replacement using Chain A from the structure of wild type *Ec*NFeoB as a search model (PDB code 3HYR, residues 1-260). Selenomethionine residues were replaced with methionine and all water molecules were removed from the model prior to the molecular replacement. Molecular replacement was performed with PHASER [4] using diffraction data to 2.1 Å resolution for S150A, and 2.8 Å for T151A. The resulting models underwent rigid body refinement followed by restrained refinement with REFMAC5 [5] and Phenix [6]. Model building, and placement of ligands and water molecules was carried out in COOT [7]. Coordinates and structure factors were deposited to the Protein Data Bank under codes 4Q00 (apo-S150A) and 4Q5I (apo-T151A).

Structural description of apo-S150A and apo-T151A

Overall, the mutants closely resembled the wild type structure, with the C_α positions superimposing with an r.m.s.d of ~0.5 for all the mutants. As inferred from the structural similarity, the domains and overall features were as previously described for the wild type protein [8]. Briefly, *Ec*NFeoB is divided into two clearly distinguished domains; the N-terminal GTP binding domain (residues 1-166) has a typical Ras-like fold consisting of a 7-stranded β sheet flanked by 6 α helices, harboring the G1-5 motifs and the effector binding regions (Switch I and II). The C-terminal 'helical' domain (residues 178-270) forms a α helical bundle comprising 5 α helices distal to the nucleotide-binding pocket.

The structure for the S150A mutant protein was solved to a resolution of 2.1 Å in the space group $P2_1$. Unlike the previously described structures for *Ec*NFeoB, the S150A structure crystallized as a monomer with three molecules per asymmetric unit. The G5 loop takes a conformation virtually identical to that of the apo-wild type structure (Figure 4A), which is conformationally distinct from its nucleotide-bound form (Figure 4B). In the wild type structure, the bound nucleotide is stabilized by electrostatic interactions between the S150 backbone amide, O6 atom of the nucleotide, and the carboxamide group of N120. In addition, the S150 hydroxyl group forms a hydrogen bond with Asn120, an electrostatic interaction that has previously been shown to cause steric crowding and destabilization of the nucleotide base [9,10]. This interaction is lost in the S150A mutant protein, which explains the increased nucleotide affinity and decreased propensity to release GDP.

The crystal structure of the apo-T151A mutant was refined to 2.8 Å resolution with R and R_{free} values of 0.233 and 0.291, respectively. Apo-T151A crystallized in the space group P_1 with 6 molecules per asymmetric unit. In the T151A structure, the G5 loop is oriented in the 'out' conformation, away from the nucleotide-binding site and shows an almost identical conformation to the nucleotide free wild type structure (Figure 4C), but structurally distinct from the wild type nucleotide bound structure (Figure 4D). In the wild type structure, the T151 hydroxyl group participates in electrostatic interactions with the N1 nitrogen atom of the nucleotide (3.3 Å distance), whilst the methyl group of the residue further stabilizes the nucleotide via hydrophobic interactions. These stabilizing interactions are eliminated in the T151A mutant, which explains the significant change in GDP release rate and affinity.

Supplementary References

1 Leslie, A. G. W. and Powell, H. R. (2007) Processing Diffraction Data with Mosflm, In Evolving Methods for Macromolecular Crystallography, pp. 41-51

2 Winn, M. D., Ballard, C. C., Cowtan, K. D., Dodson, E. J., Emsley, P., Evans, P. R., Keegan, R. M., Krissinel, E. B., Leslie, A. G., McCoy, A., McNicholas, S. J., Murshudov, G. N., Pannu, N. S., Potterton, E. A., Powell, H. R., Read, R. J., Vagin, A. and Wilson, K. S. (2011) Overview of the CCP4 suite and current developments. Acta Crystallogr D Biol Crystallogr **67**, 235-242

3 Otwinowski, Z., Minor, W. and Charles W. Carter, J. (1997) [20] Processing of X-ray diffraction data collected in oscillation mode, In Methods in Enzymology Macromolecular Crystallography Part A, pp. 307-326, Academic Press

4 McCoy, A. J., Grosse-Kunstleve, R. W., Adams, P. D., Winn, M. D., Storoni, L. C. and Read, R. J. (2007) Phaser crystallographic software. Journal of Applied Crystallography **40**, 658-674

5 Murshudov, G. N., Skubák, P., Lebedev, A. A., Pannu, N. S., Steiner, R. A., Nicholls, R. A., Winn, M. D., Long, F. and Vagin, A. A. (2011) REFMAC5 for the refinement of macromolecular crystal structures. Acta Crystallogr D Biol Crystallogr **67**, 355-367

6 Adams, P. D., Afonine, P. V., Bunkóczi, G., Chen, V. B., Davis, I. W., Echols, N., Headd, J. J., Hung, L. W., Kapral, G. J., Grosse-Kunstleve, R. W., McCoy, A. J., Moriarty, N. W., Oeffner, R., Read, R. J., Richardson, D. C., Richardson, J. S., Terwilliger, T. C. and Zwart, P. H. (2010) PHENIX: a comprehensive Python-based system for macromolecular structure solution. Acta Crystallogr D Biol Crystallogr **66**, 213-221

7 Emsley, P. and Cowtan, K. (2004) Coot: model-building tools for molecular graphics. Acta Crystallogr D Biol Crystallogr **60**, 2126-2132

8 Guilfoyle, A., Maher, M. J., Rapp, M., Clarke, R., Harrop, S. and Jormakka, M. (2009) Structural basis of GDP release and gating in G protein coupled Fe2+ transport. EMBO J **28**, 2677-2685

9 Guilfoyle, A. P., Deshpande, C. N., Vincent, K., Pedroso, M. M., Schenk, G., Maher, M. J. and Jormakka, M. (2014) Structural And Functional Analysis Of A Feob A143s G5 Loop Mutant Explains Accelerated Gdp Release Rate. FEBS J

10 Posner, B. A., Mixon, M. B., Wall, M. A., Sprang, S. R. and Gilman, A. G. (1998) The A326S Mutant of Gialpha 1 as an Approximation of the Receptor-bound State. J. Biol. Chem. **273**, 21752-21758
